# A Simple Statistic for Comparing Moderation of Slopes and Correlations

**DOI:** 10.3389/fpsyg.2012.00231

**Published:** 2012-07-09

**Authors:** Michael Smithson

**Affiliations:** ^1^Psychology, The Australian National UniversityCanberra, ACT, Australia

**Keywords:** moderator effects, interaction effects, heteroscedasticity, regression, correlation

## Abstract

Given a linear relationship between two continuous random variables *X* and *Y* that may be moderated by a third, *Z*, the extent to which the correlation ρ is (un)moderated by *Z* is equivalent to the extent to which the regression coefficients *β_y_* and *β_x_* are (un)moderated by *Z* iff the variance ratio $\sigma_{y}^{2}∕\sigma_{x}^{2}$ is constant over the range or states of *Z*. Otherwise, moderation of slopes and of correlations must diverge. Most of the literature on this issue focuses on tests for heterogeneity of variance in *Y*, and a test for this ratio has not been investigated. Given that regression coefficients are proportional to ρ via this ratio, accurate tests, and estimations of it would have several uses. This paper presents such a test for both a discrete and continuous moderator and evaluates its Type I error rate and power under unequal sample sizes and departures from normality. It also provides a unified approach to modeling moderated slopes and correlations with categorical moderators via structural equations models.

## Introduction

Let *X* and *Y* have a bivariate normal distribution, $X\sim N\left(\mu_{x},\sigma_{x}^{2}\right),$ and $Y\sim N\left(\mu_{y},\sigma_{y}^{2}\right).$ Suppose also that the correlation between *X* and *Y* is a function of a moderator variable *Z*. Under homogeneity of variance (HoV), moderation of correlations implies moderation of regression coefficients (or means, in ANOVA), and vice versa. For example, establishing the existence of a moderator effect from *Z* in a linear regression model with *X* and *Z* predicting *Y* by finding a significant regression coefficient for the product term *X* × *Z* suffices to infer a corresponding moderator effect of *Z* on the correlation between *X* and *Y*.

Heterogeneity of variance (HeV) due to *Z*, however, can alter moderator effects so that correlation and regression coefficients are not equivalently moderated. We may have moderation of slopes, for instance, without moderation of correlations, moderation of correlations with no moderation of slopes, moderation of slopes and correlations in opposite directions, or even moderation of regression coefficients in opposite directions (e.g., what appears to be a positive moderator effect when *X* predicts *Y* becomes a negative effect when *Y* predicts *X*).

Although some scholars have warned about the impacts of heteroscedasticity on the analysis of variance (e.g., Grissom, 2000) and linear regression, most contemporary textbook advice and published evidence on this matter comforts researchers with the notion that ANOVA and regression are fairly robust against it. Howell (2007, p. 316), for instance, states that despite Grissom’s pessimistic outlook “the homogeneity of variance assumption can be violated without terrible consequences” and advises that for symmetrically distributed populations and equal numbers of participants in each cell, the validity of ANOVA is likely if the ratio of the largest to the smallest variance is no greater than 4. Tabachnick and Fidell (2007, pp. 121–123) are even more relaxed, recommending an upper limit on this ratio of 10 before raising an alarm. A recent investigation into the robustness of one-way ANOVA against violations of normality (Schmider et al., 2010) also is relatively reassuring on that count. A fairly recent comparison of several tests of homogeneity of variance (Correa et al., 2006) generally finds in favor of the Levene test but leaves the issue of the impact of HoV on moderator effects unexamined.

Nevertheless, this problem is well-known. Arnold (1982) drew a distinction between the “form” and “degree” of moderator effects, whereby the “form” is indexed by moderation of slopes (or means, in ANOVA) whereas the “degree” is indexed by moderation of correlations. He argued from first principles and demonstrated empirically that it is possible to find a significant difference between correlations from two independent-samples but fail to find a corresponding significant regression interaction term, and vice versa. A related treatment was presented independently by Sharma et al. (1981), who referred to “degree” moderators as “homologizers” (a term taken from Zedeck, 1971). They pointed out that homologizers that act through the error-term in a regression instead of through the predictor itself.

Stone and Hollenbeck (1984) dissented from Arnold (1982), arguing that only moderated regression is needed to assess moderating effects, regardless of whether they are of form or degree. Their primary claims were that moderated slopes also can be interpreted as differing strengths of relationship, and that the subgrouping method advocated by Arnold raises concerns about how subgroups are created if the moderator is not categorical. Arnold (1984) rebutted their claim regarding the slope as a measure of relationship strength, reiterating the position that slopes, and correlations convey different types of information about such relationships. He also declared that both moderated regression and tests of differences between correlation coefficients are essentially “subgroup” methods. At the time there was no way to unify the examination of moderation of correlations and slopes. The present paper describes and demonstrates such an approach for categorical moderators, via structural equations models.

In a later paper, Stone and Hollenbeck (1989) reprised this debate and recommended variance-stabilizing and homogenizing transformations as a way to eliminate the apparent disagreement between moderation of correlations and moderation of slopes. These include not only transformations of the dependent variable, but also within-groups standardization and/or normalization. They also, again, recommended abandoning the distinction between degree and form moderation and focusing solely on form (i.e., moderated regression). The usual cautions against routinely transforming variables and objections to applying different transformations to subsamples aside, we shall see that transforming the dependent variable is unlikely to eliminate the non-equivalence between moderation of slopes and correlations. Moreover, other investigators of this issue do not arrive at the same recommendation as Stone and Hollenbeck when it comes to a “best” test.

Apparently independently of the aforementioned work, and extending the earlier work of Dretzke et al. (1982), Alexander and DeShon (1994) demonstrated severe effects from heterogeneity of error-variance (HeEV) on power and Type I error rates for the F-test of equality of regression slopes. In contrast to Stone and Hollenbeck (1989), they concluded that for a categorical moderator, the “test of choice” is the test for equality of correlations across the moderator categories, provided that the hypotheses of equal correlations and equal slopes are approximately identical.

These hypotheses are equivalent if and only if the ratio of the variance in *X* to the variance in *Y* is equal across moderator categories (Arnold, 1982; Alexander and DeShon, 1994). The reason for this is clear from the textbook equation between correlations and unstandardized regression coefficients. For the *i*th category of the moderator,

(1) $$\beta_{yi}=\rho_{i}\frac{\sigma_{yi}}{\sigma_{xi}}$$

For example, a simple algebraic argument shows that if the σ*_yi_/*σ*_xi_* ratio is not constant for, say, *i* = 1 and *i* = 2 then *β*_1_ = *β*_2_ ⇒ *ρ*_1_ ≠ *ρ*_2_, and likewise *ρ*_1_ = *ρ*_2_ ⇒ *β*_1_ ≠ *β*_2_. More generally,

(2) $$\frac{\sigma_{y1}\sigma_{x2}}{\sigma_{x1}\sigma_{y2}}>\left(<\right)1\Leftrightarrow \left|\frac{\beta_{1}}{\beta_{2}}\right|>\left(<\right)\left|\frac{\rho_{1}}{\rho_{2}}\right|.$$

The condition for correlations and slopes to be moderated in opposite directions follows immediately: *β*_1_ > *β*_2_ but *ρ*_2_ > *ρ*_1_ if when *ρ*_2_ > *ρ*_1_, it is also true that

$$\frac{\sigma_{y1}\sigma_{x2}}{\sigma_{x1}\sigma_{y2}}>\frac{\rho_{2}}{\rho_{1}}.$$

The same implication holds if the inequalities are changed from > to <.

The position taken in this paper is that in multiple linear regression there are three distinct and valid types of moderator effects. First, in multiple regression equation (1) generalizes to a version where standardized regression coefficients replace correlation coefficients:

(3) $$\beta_{yi}=B_{yi}\frac{\sigma_{yi}}{\sigma_{xi}}$$

where *B_yi_* is a standardized regression coefficient. Thus, we have moderation of unstandardized versus standardized regression coefficients (or correlations when there is only one predictor), which are equivalent if and only if the aforementioned variance ratio is equal across moderator categories. Otherwise, the assumption that moderation of one implies equivalent moderation of the other is mistaken. This is a simple generalization of Arnold’s (1982) and Sharma et al.’s (1981) distinction.

Second, the semi-partial correlation coefficient, ν*_xi_*, is a simple function of *B_yi_* and tolerance. In the *i*th moderator category, the tolerance of a predictor, *X*, is $T_{xi}=1-R_{xi}^{2},$ where $R_{xi}^{2}$ is the squared multiple correlation for *X* regressed on the other predictors included in the multiple regression model. The standardized regression coefficient, semi-partial correlation, and tolerance are related by

$$\nu_{xi}=B_{yi}\sqrt{T_{xi}}.$$

Equation (3) therefore may be rewritten as

(4) $$\beta_{yi}=\nu_{xi}\frac{\sigma_{yi}}{\sigma_{xi}\sqrt{T_{xi}}}.$$

Thus, we have a distinction between the moderation of the unique contribution of a predictor to the explained variance of a dependent variable and moderation of regression coefficients (whether standardized or not). Equivalence with moderation of standardized coefficients (or simple correlations) hinges on whether tolerance is constant across moderator categories (an issue not dealt with in this paper), while equivalence with moderation of unstandardized coefficients depends on both constant tolerance and constant variance ratios.

In a later paper, DeShon and Alexander (1996) proposed alternative procedures for testing equality of regression slopes under HeEV, but they and both earlier and subsequent researchers appear to have neglected the idea of testing for equal variance ratios (EVR) across moderator categories. This is understandable, given that HeEV is a more general concern in some respects and the primary object of most regression (and ANOVA) models is prediction.

Nevertheless, it is possible for HeEV to be satisfied when EVR is not. An obvious example is when there is HoV for *Y* and equality of correlations across moderator categories but HeV for *X*. These conditions entail HeEV but also imply that slopes cannot be equal across categories. This case seems to have been largely overlooked in the literature on moderators. More generally, HeEV is ensured when, for all *i* and *j*,

(5) $$\frac{\sigma_{yi}^{2}}{\sigma_{yj}^{2}}=\frac{1-\rho_{j}^{2}}{1-\rho_{i}^{2}},$$

which clearly has no bearing on whether EVR holds or not.

Thus, a test of EVR would provide a guide for determining when equality of slopes and equality of correlations are equivalent null hypotheses and when not. Given that it is not uncommon for researchers to be interested in both moderation of slopes (or means) and moderation of correlations, this test could be a useful addition to data screening procedures.

It might seem that if researchers are going to test for both moderation of slopes and correlations, a test of EVR is superfluous. However, the joint outcome of the tests of equal correlations and equal slopes does not render the question of EVR moot or irrelevant. The reason this should interest applied researchers is that the tests of equal correlations and equal slopes will not inform them of whether the moderation of slopes is equivalent to the moderation of correlations, whereas a test of EVR would do exactly that. Suppose, for example, the test for equality of slopes yields *p* = 0.04 (so we reject the null hypothesis) whereas the corresponding test for correlations yields *p* = 0.06 (so we fail to reject). An EVR test would tell us whether these two outcomes are genuinely unequal or whether their apparent difference may be illusory. Thus, an EVR test logically should take place *before* tests of equality of slopes or correlations, because it will indicate whether both of the latter tests need to be conducted or just one will suffice.

Furthermore, an estimate of the ratio of the variance ratios along with its standard error provides an estimate of (and potentially a confidence interval for) a ratio comparison between moderation of slopes and moderation of correlations. From equations (1) and (2), for the *i*th and *j*th moderator categories, we immediately have

(6) $$\frac{\sigma_{yi}∕\sigma_{xi}}{\sigma_{yj}∕\sigma_{xj}}=\frac{\beta_{yi}∕\beta_{yj}}{\rho_{i}∕\rho_{j}}.$$

Finally, equation (3) tells us that an EVR test can be used to assess the equivalence between the moderation of standardized and unstandardized regression coefficients, thereby expanding its domain of application into multiple regression.

All said and done, it is concerning that numerous articles in the foremost journals in psychology routinely report tests of interactions in ANOVAs, ANCOVAs, and regressions with no mention of prior testing for either HeV or HeEV. Moreover, reviews of the literature on metric invariance by Vandenberg and Lance (2000) and DeShon (2004) indicated considerable disagreement on the importance of HeEV for assessments of measurement invariance across samples in structural equations models. Researchers are unlikely to be strongly motivated to use a test for EVR unless it is simple, readily available in familiar computing environments, robust, and powerful. We investigate such a test with these criteria in mind.

## A Test of EVR for Categorical Moderators

An obvious candidate for a test of EVR is a parametric test based on the log-likelihood of a bivariate normal distribution for *X* and *Y* conditional on a categorical moderator *Z*. We employ submodels for the standard deviations using the log link. Using the first category of the moderator as the “base” category, the submodels may be written as

$$\begin{array}{rcll} \sigma_{xi}=\text{exp}\left(\underset{i}{\sum }\;z_{i}\delta_{xi}\right), & & & \text{(7)}\\ \sigma_{yi}=\text{exp}\left(\underset{i}{\sum }\;z_{i}\delta_{yi}\right), & & & \end{array}$$

where *z*_1_ = 1 and for *i* > 1 *z_i_* is an indicator variable for the *i*th category of *Z*, and the δ parameters are regression coefficients. Under the hypothesis that EVR holds between the *i*th and first categories, the relevant test statistic is

(8) $$\theta_{i}=\delta_{yi}-\delta_{xi},$$

for *i* > 1, with

(9) $$\text{var}\left(\theta_{i}\right)=\text{var}\left(\delta_{yi}\right)+\text{var}\left(\delta_{xi}\right)-2\text{cov}\left(\delta_{yi},\delta_{xi}\right),$$

and the assumption that δ*_yi_* and δ*_yi_* are asymptotically bivariate normally distributed. Immediately we have a confidence interval for θ*_i_*, namely ${\hat{\theta }}_{i}\alpha t_{\alpha ∕2}\sqrt{\text{v}\hat{\text{ar}}}\left(\theta_{i}\right),$ where *t*_α/2_ is the 1-α/2 quantile of the t distribution with the appropriate degrees of freedom for an independent-samples test. We also have

(10) $$\exp\left(\theta_{i}\right)=\frac{\beta_{yi}/\beta_{y1}}{\rho_{i}/\rho_{1}},$$

and we may exponentiate the limits of this confidence interval to obtain a confidence interval for the right-hand expression in this equation, i.e., for the ratio comparison between the ratio of moderated regression coefficients and the ratio of moderated correlations.

The hypothesis that θ*_i_* = 0 is equivalent to a restricted model in which, for *i* > 1, δ*_xi_* = δ*_yi_*. The modeling approaches outlined later in this paper make use of this equivalence. More complex EVR hypotheses may require different design matrices from the setup proposed in this introductory treatment. First, however, we shall examine the properties of θ, including Type I error rates and power under unequal sample sizes, and the effects of departures from normality for *X* and *Y*.

## Assessing Type I Error Accuracy and Power

We begin with simulations testing null hypothesis rejection rates for EVR when the null hypotheses of EVR and unmoderated correlations and slopes are true. Simulations using a two-category moderator (20,000 runs for each condition) were based on DeShon and Alexander, 1996; Table 1), with constant variance ratio of 2, $\rho_{xy}=1∕\sqrt{2}$, and β*_y_* = 1 for both categories. Three pairs of sample sizes were used (again based on DeShon and Alexander, 1996): 70 for both samples, 45 for one and 155 for the second, and 90 for one and 180 for the second. Three pairs of variances also were used, to ascertain any impact from the sizes of the variances. All runs used a Type I error criterion of α = 0.05.

**Table 1:** **Type I error: two-groups simulations**.

Skew	*N*_1_	*N*_2_	σ*_xi_* = 1,1	σ*_xi_* = 1,2	σ*_xi_* = 1,4
			σ*_yi_* = 2,2	σ*_yi_* = 2,4	σ*_yi_* = 2,8
**NORMAL**					
0	70	70	0.0518	0.0532	0.0511
0	45	155	0.0578	0.0553	0.0547
0	90	180	0.0513	0.0531	0.0503
**SKEWED**					
2	70	70	0.0710	0.0704	0.0680
4	70	70	0.0768	0.0767	0.0713
2	45	155	0.0681	0.0686	0.0687
4	45	155	0.0778	0.0776	0.0774
2	90	180	0.0679	0.0708	0.0714
4	90	180	0.0755	0.0728	0.0723

The top half of Table 1 shows the EVR rejection rates for random samples from normally distributed *X* and *Y*. Unequal sample sizes have little impact on rejection rates, with the effect appearing to diminish in the larger-sample (90–180) condition. The rates are slightly higher than 0.05, but are unaffected by the sizes of the variances.

The lower half of Table 1 shows simulations under the same conditions, but this time with *X* and *Y* sampled from the Azzalini skew-normal distribution (Azzalini, 1985). The standard skew-normal pdf is

$$f\left(x,\lambda \right)=\frac{e^{-x^{2}}/2}{\sqrt{2\pi }}\left(1+Erf\left[\frac{\lambda x}{\sqrt{2}}\right]\right).$$

**Figure 1:**
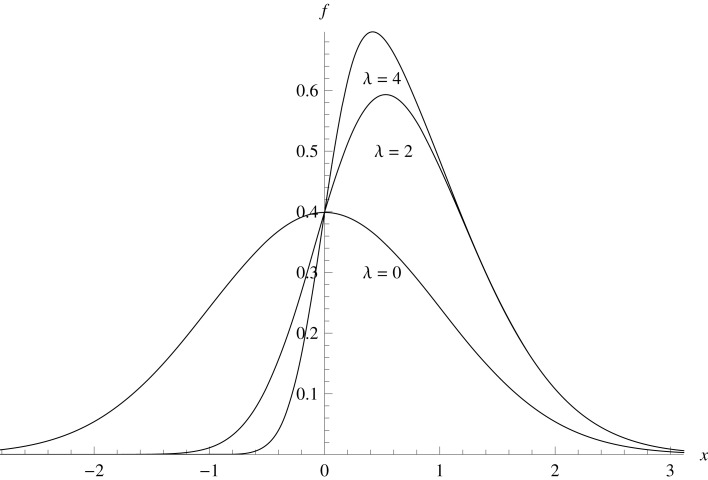
**Azzalini Skew-normal distributions with λ = 0,2,4**.

The simulations had the skew parameter λ set to 2 and 4, the pdfs for which are shown in Figure 1. Skew increased the rejection rates to 0.068–0.078, rendering the test liberal but not dramatically so.

We now turn to investigating the power of the EVR test. Simulations testing its power were conducted for two situations: moderated slopes but unmoderated correlations, and moderated correlations but unmoderated slopes. Both batches of simulations were run with four combinations of sample sizes (70–70, 40–140, 140–140, and 80–280) and three variance ratio combinations (1–1.5, 1–2, 1–4). In the unmoderated correlations setup ρ = 0.5 for all conditions, and in the unmoderated slopes setup β*_y_* = 0.5 for all conditions. These tests also require modeling the moderation of correlations. The correlation submodel uses the Fisher link, i.e.

(11) $$\text{log}\left(\frac{1+\rho_{i}}{1-\rho_{i}}\right)=\underset{i}{\sum }\;w_{i}\delta_{ri}.$$

Note that we allow a different set of predictors for the correlation from those in equation (7). However, in this paper we will impose the restriction *w_i_* = *z_i_*.

Table 2 shows the simulation results for unequal variance ratios with unmoderated correlations. The table contains rejection rates of the EVR and moderation of correlation null hypotheses. The resultant moderated slopes and error-variances are displayed for each condition. Note that HeV and HeEV do not have discernible effects on either of the rejection rates. As in the preceding simulations, the rejection rates for the unmoderated correlations are only slightly above the 0.05 criterion. The rejection rates for the EVR test l and in the 0.85–1.0 range in the conditions where the combined sample sizes are 280 and the ratio of the variance ratios is 2:1 or for both combined sizes when the ratio is 4:1.

**Table 2:** **Power: moderated slopes and unmoderated correlations**.

*N*_1_	*N*_2_	$\sigma_{x}^{2}=2$	$\sigma_{x}^{2}=2$	$\sigma_{x}^{2}=2$	$\sigma_{x}^{2}=2$	$\sigma_{x}^{2}=2$	$\sigma_{x}^{2}=2$
		$\sigma_{y}^{2}=2$	$\sigma_{y}^{2}=3$	$\sigma_{y}^{2}=2$	$\sigma_{y}^{2}=4$	$\sigma_{y}^{2}=2$	$\sigma_{y}^{2}=8$
		σ*_xy_* = 1	$\sigma_{xy}=\sqrt{3∕2}$	σ*_xy_* = 1	$\sigma_{xy}=\sqrt{2}$	σ*_xy_* = 1	σ*_xy_* = 2
		$\sigma_{e}^{2}=1.5$	$\sigma_{e}^{2}=2.25$	$\sigma_{e}^{2}=1.5$	$\sigma_{e}^{2}=3$	$\sigma_{e}^{2}=1.5$	$\sigma_{e}^{2}=\sqrt{8}∕2$
		β*_y_* = 0.5	$\beta_{y}=\sqrt{3∕8}$	β*_y_* = 0.5	$\beta_{y}=\sqrt{2}∕2$	β*_y_* = 0.5	β*_y_* = 1
		δ*_r_*	θ	δ*_r_*	θ	δ*_r_*	θ
70	70	0.0556	0.2810	0.0603	0.6321	0.0576	0.9939
40	100	0.0566	0.2478	0.0569	0.5706	0.0566	0.9875
140	140	0.0549	0.4841	0.0532	0.9032	0.0537	1.000
80	200	0.0529	0.4311	0.0497	0.8524	0.0522	0.9999

Table 3 shows the rejection rates of the EVR and moderation of correlation null hypotheses when there are unequal variance ratios and moderated correlations. The resultant moderated correlations and error-variances are displayed for each condition. As before, HeV and HeEv do not affect either of the rejection rates. Likewise, as expected, the EVR rejection rates are very similar to those in Table 2. It is noteworthy that rejection rates for the unmoderated correlations hypothesis are considerably smaller than those for the EVR hypothesis, even though the correlations differ fairly substantially. It is well-known that tests for moderation of slopes and correlations have rather low power. These results, and the fact that the ratios of the variance ratios do not exceed Howell’s benchmark of 4:1, suggest that the EVR test has relatively high power.

**Table 3:** **Power: unmoderated slopes and moderated correlations**.

*N*_1_	*N*_2_	$\sigma_{x}^{2}=2$	$\sigma_{x}^{2}=2$	$\sigma_{x}^{2}=2$	$\sigma_{x}^{2}=2$	$\sigma_{x}^{2}=2$	$\sigma_{x}^{2}=2$
		$\sigma_{y}^{2}=2$	$\sigma_{y}^{2}=3$	$\sigma_{y}^{2}=2$	$\sigma_{y}^{2}=4$	$\sigma_{y}^{2}=2$	$\sigma_{y}^{2}=8$
		σ*_xy_* = 1	σ*_xy_* = 1	σ*_xy_* = 1	σ*_xy_* = 1	σ*_xy_* = 1	σ*_xy_* = 1
		$\sigma_{y}^{2}=1.5$	$\sigma_{y}^{2}=2.5$	$\sigma_{y}^{2}=1.5$	$\sigma_{y}^{2}=3.5$	$\sigma_{y}^{2}=1.5$	$\sigma_{e}^{2}=6$
		ρ*_xy_* = 0.5	$\rho_{xy}=1/\sqrt{6}$	ρ*_xy_* = 0.5	$\rho_{xy}=1/\sqrt{8}$	ρ*_xy_* = 0.5	ρ*_xy_* = 0.25
		δ*_r_*	θ	δ*_r_*	θ	δ*_r_*	θ
70	70	0.0944	0.2635	0.1525	0.5925	0.3426	0.9864
40	100	0.0992	0.2394	0.1700	0.5476	0.3558	0.9826
140	140	0.1296	0.4575	0.2483	0.8771	0.5844	1.000
80	200	0.1444	0.4201	0.2878	0.8326	0.6036	0.9999

## Structural Equations Model Approach

**Figure 2:**
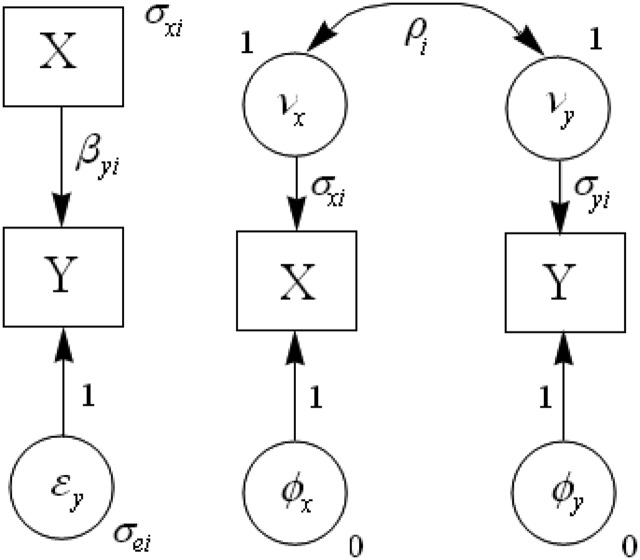
**Moderated regression and correlation structural equations models**.

When the moderator variable is categorical, the EVR test can be incorporated in a structural equations model (SEM) approach that permits researchers not only to compare an EVR model against one that relaxes this assumption, but also to test simultaneously for HeV, HeEV, moderation of correlations and moderation of slopes. Figure 2 shows the regression (left-hand side) and correlation (right-hand side) versions of this model. The latter follows Preacher’s (2006) strategy for a multi-group SEM for correlations. The regression version models the error-variances $\sigma_{ei}^{2}$ rather than the variances $\sigma_{yi}^{2}.$ Instead, $\sigma_{yi}^{2}$ is modeled in the correlation version. The only addition to the correlation SEM required for incorporating EVR tests is to explicitly model variance ratios for each of the moderator variable categories. Two SEM package that can do so are lavaan (Rosseel, 2012) in R and MPlus (Muthén and Muthén, 2010). Examples in lavaan and MPlus are available at http://dl.dropbox.com/u/1857674/EVR_moderator/EVR.html, as are EVR test scripts in SPSS and SAS.

Simulations were run using lavaan in model comparisons for samples with moderated slopes but unmoderated correlations, and samples with moderated correlations but unmoderated slopes. As before, each simulation had 20,000 runs.

Simulations from bivariate normal distributions with ρ*_xy_* = 0.05 for both groups (Table 4) indicated that moderately large samples and slope differences are needed for reasonable power. However, there was little impact on power from unequal group sizes. Rejection rates for the unmoderated correlations hypothesis were at appropriate levels, 0.0493–0.0559.

**Table 4:** **Moderated regression coefficients**.

*N*_1_	*N*_2_	β*_y_* = 0.50	β*_y_* = 0.50	β*_y_* = 0.50
		β*_y_* = 0.61	β*_y_* = 0.71	β*_y_* = 1.00
70	70	0.1086	0.2030	0.5659
40	100	0.1012	0.2026	0.6031
140	140	0.1633	0.3668	0.8566
80	200	0.1499	0.3549	0.8875

Likewise, simulations from bivariate normal distributions with β*_y_* = 0.5 for both groups (Table 5) indicated that moderately large samples and correlation differences are needed for reasonable power. There was a slight to moderate impact from unequal group sizes, somewhat greater than the impact in Table 4. Rejection rates for the unmoderated slopes hypothesis were appropriately 0.0484–0.0538.

**Table 5:** **Moderated correlations**.

*N*_1_	*N*_2_	ρ*_xy_* = 0.50	ρ*_xy_* = 0.50	ρ*_xy_* = 0.50	ρ*_xy_* = 0.50
		ρ*_xy_* = 0.41	ρ*_xy_* = 0.35	ρ*_xy_* = 0.25	ρ*_xy_* = 0.17
70	70	0.1159	0.1984	0.4406	0.6390
40	100	0.1031	0.1728	0.3610	0.5487
140	140	0.1760	0.3521	0.7215	0.9008
80	200	0.1541	0.2960	0.6312	0.8395

## SEM Example

Consider a population with two normally distributed variables *X*, political liberalism, and *Y*, degree of belief in global warming. Suppose that they are measured on scales with means of 0 and standard deviations of 1, and the correlation between these two scales is ρ = 0.45. Suppose also that if members of this population are exposed to a video debate highlighting the arguments for and against the reality of global warming, it polarizes belief in global warming by increasing the degree of belief of those who already tend to believe it and decreasing the degree of belief of those who already are skeptical. Thus, the standard deviation doubles from 1 to 2. However, the mean remains at 0 and the correlation between belief in global warming and political liberalism also is unchanged, remaining at 0.45.

In a two-condition experiment with half the participants from this population assigned to a condition where they watch the video and half to a “no-video” condition, the experimental conditions may be regarded as a two-category moderator variable *Z*. We have ρ = 0.45 and σ*_x_* = 1 regardless of *Z*, and σ*_y1_* = 2 whereas σ*_y2_* = 1. It is also noteworthy that when *X* predicts *Y* HeEV is violated whereas when *Y* predicts *X* it is not.

We randomly sample 600 people from this population and randomly assign 300 to each condition, representing the video condition with *Z* = 1 and the no-video condition with *Z* = −1. As expected, the sample correlations in each subsample do not differ significantly: *r*_1_ = 0.458, *r*_2_ = 0.463, and Fisher’s test yields *z* = 0.168 (*p* = 0.433). However, a linear regression with *Y* predicted by *X* and *Z* that includes an interaction term (*Z*  × *X*) finds a significant positive interaction coefficient (*z* = 3.987, *p* < 0.0001). Taking the regression on face value could mislead us into believing that because the slope between *X* and *Y* differs significantly between the two categories of *Z*, *Z* also moderates the association between *X* and *Y*. Of course, it does not. Seemingly more puzzling is the fact that linear regression with *Y* predicting *X* yields a significant *negative* interaction term (*Z*  × *Y*) with *z* = −3.859 (*p* = 0.0001). So the regression coefficient is moderated in opposite directions, depending on whether we predict *Y* or *X*.

**Figure 3:**
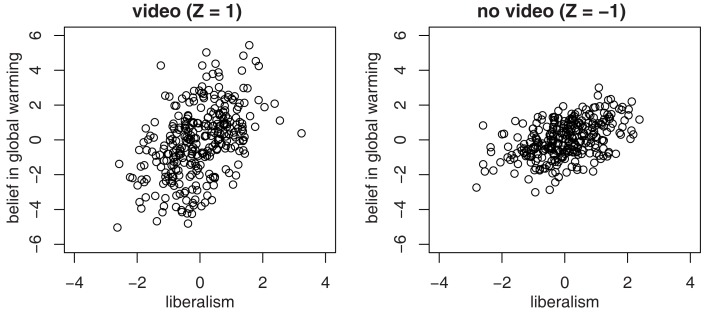
**Scatter plots for the two-condition experiment**.

The scatter plots in Figure 3 provide an intuitive idea of what is going on. Clearly the slope for *Y* (belief in global warming) predicted by *X* (liberalism) appears steeper when *Z* = 1 than when *Z* = −1. Just as clearly, the slope for *X* predicted by *Y* appears less steep when *Z* = 1 than when *Z* = −1. The oval shapes of the data distribution in both conditions appear similar to one another, giving the impression that the correlations are similar.

We now demonstrate that the SEM approach can clarify and validate these impressions, using Mplus 6.12. We begin with the moderation of slopes models. Because σ*_x1_* = σ*_x2_* (i.e., *X* has HoV) we may move from the saturated model to one that restricts those parameters to be equal. The model fit is χ^2^(1) = 0.370 (*p* = 0.543). This baseline model also reproduces the slopes estimates in OLS regression. Now, a model removing HoV for *X* and imposing the EVR restriction yields χ^2^(1) = 82.246 (*p* < 0.0001), so clearly we can reject the EVR hypothesis. Fitting another model with HoV in *X* and HeV in *Y* but where we set β*_y1_* = β*_y2_*, the fit is χ^2^(2) = 15.779 (*p* = 0.0004), and the model comparison test is χ^2^(1) = 15.779 − 0.370 = 15.409 (*p* < 0.0001). We conclude there is moderation of slopes but EVR does not hold, so we expect that the moderation of correlations will differ from that of the slopes, and the moderation of slopes will differ when *X* predicts *Y* versus when *Y* predicts *X*. Indeed, if we fit models with *Y* predicting *X* we also can reject the equal slopes model, and the slopes differ in opposite directions across the categories of *Z*. When *X* predicts *Y* β*_y1_* = 0.496 and β*_y2_* = 0.978, whereas when *Y* predicts *X* β*_x1_* = 0.219 and β*_x2_* = 0.423.

Turning to correlations, we start with a model that sets σ*_x1_* = σ*_x2_* (i.e., assuming that *X* has HoV) and leaves all other parameters free. The fit is χ^2^(1) = 0.370 (*p* = 0.543), identical to the equivalent baseline model described above. This model closely reproduces the sample correlations (the parameter estimates are 0.452 and 0.469, versus the sample correlations 0.458 and 0.463). Moreover, a model adding the EVR restriction yields χ^2^(1) = 82.246, again identical to the equivalent regression model. Now if we set ρ_1_ = ρ_2_, the fit is χ^2^(2) = 0.453 (*p* = 0.797) and the model comparison test is χ^2^(1) = 0.083 (*p* = 0.773). Thus, there is moderation of slopes but not of correlations.

## Continuous Moderators

Continuous moderators pose considerably greater challenges than categorical ones, because of the many forms that HeV and HeEV can take. Arnold (1982) sketched out a treatment of this problem that is not satisfactory, namely correlating correlations between *X* and *Y* with values of the continuous moderator *Z*. In an innovative paper, Allison et al. (1992) extended a standard approach to assessing heteroscedasticity to test for homologizers when the moderator variable, Z, is continuous. Their technique is simply to compute the correlation between Z and the absolute value of the residuals from the regression equation that already includes both the main effect for Z and the interaction term. This is a model of moderated error, akin to modeling error-variance, which is useful in itself but not equivalent to testing for EVR. In their approach and the simulations that tested it, Allison et al. assumed HeV for their predictor, thereby ignoring the fact that EVR can be violated even when HeEV is satisfied.

The approach proposed here generalizes the model defined by equations (7) and (11), with the *z_i_* now permitted to be continuous. This model is

(12) $$\begin{array}{l} \log\left(\sigma_{x}\right)=\sum_{i}z_{i}\delta_{xi},\\ \log\left(\sigma_{y}\right)=\sum_{i}z_{i}\delta_{yi},\\ \log\left(\frac{1+\rho_{xy}}{1-\rho_{xy}}\right)=\sum_{i}z_{i}\delta_{ri}, \end{array}$$

where *z*_1_ = 1 and for *i* > 1 the *z_i_* are continuous random variables. The δ*_xi_*, δ*_yi_*, and δ*_ri_* coefficients can be simultaneously estimated via maximum likelihood, using the likelihood function of a bivariate normal distribution conditioned by the *z_i_*. Scripts for maximum likelihood estimation in R, SPSS, and SAS are available via the link cited earlier. This model can be made more flexible by introducing polynomial terms in the *z_i_*, but we do not undertake that extension here.

To begin, simulations (20,000 runs each) for a single-moderator model took samples for *Z* from a *N*(0, 1) population. *X* and *Y* were sampled from bivariate normal distributions with δ*_r1_* = 0, δ*_x1_* = δ*_y1_* =  {0, 0.5, 1.0}, and *δ_r0_* = {0, 0.5, 1.0}. Table 6 displays their results. Rejection rates are somewhat too high for δ*_r1_* but only slightly too high for θ_1_ unless sample sizes are over 200 or so.

**Table 6:** **Unmoderated continuous moderator simulations**.

*N*	δ*_r0_* = 0.0	δ*_r0_* = 0.5	δ*_r0_* = 1.0
**δ*_r1_***			
70	0.0715	0.0712	0.0685
140	0.0619	0.0589	0.0571
280	0.0543	0.0554	0.0545
**θ*_1_***			
70	0.0610	0.0627	0.0616
140	0.0548	0.0564	0.0556
280	0.0533	0.0536	0.0528

Simulations also were run under the same conditions as Table 6 but with samples from a skew-normal distribution with skew parameter λ = 2. These results are shown in Table 7. There, it can be seen that Type I error rates are inflated by skew almost independently of sample size, much more so for δ*_r1_* than θ_1_. Both are affected by size of the correlation’s moderation effect.

**Table 7:** **Simulations from Azzalini distribution with λ = 2**.

*N*	δ*_r0_* = 0.0	δ*_r0_* = 0.5	δ*_r0_* = 1.0
**δ*_r1_***			
70	0.0724	0.0874	0.1001
140	0.0682	0.0801	0.0925
280	0.0665	0.0767	0.0930
**θ*_1_***			
70	0.0554	0.0673	0.0679
140	0.0519	0.0689	0.0645
280	0.0514	0.0676	0.0636

**Figure 4:**
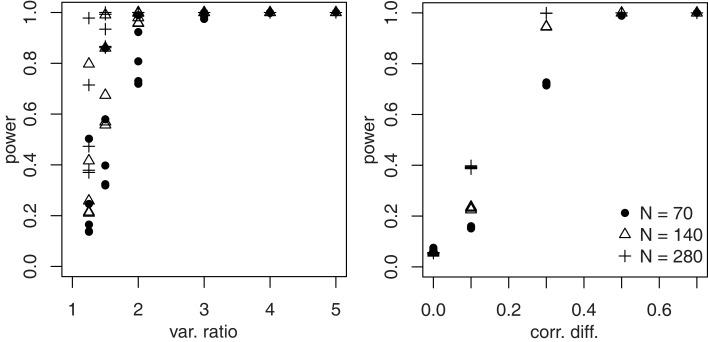
**Power for δ*_r1_* and θ*_r1_***.

To investigate power, simulations were run with δ*_r1_* = {0, 0.2007, 0.6190, 1.0986, 1.7346} (correlation differences of {0,0.1,0.3,0.5,0.7} when *z* = 1) and θ_1_ = {0.1116, 0.2027, 0.3466, 0.5493, 0.6931, 0.8047} (variance ratios of {1.25, 1.5, 2, 3, 4, 5} when *z* = 1). Thus, there were 30 simulations for each of three sample sizes (70,140, and 280). The results are displayed in Figure 4. Power for θ_1_ attains high levels even for moderate sample sizes when the variance ratio is 2 or more. However, power also is higher the more strongly correlations are moderated, whereas power for δ*_r1_* is unaffected by moderation of the variance ratio. Power for δ*_r1_* does not become high unless correlations differ by at least 0.3, and the results for a correlation difference of 0.1 are in line with those for categorical moderators (see Table 3).

The simulation results were examined for evidence of estimation bias. Both ${\hat{\delta }}_{r1}$ and θ_1_ were slightly biased upward, and most strongly for smaller samples and larger effect-sizes. The maximum average bias for ${\hat{\delta }}_{r1}$ and ${\hat{\theta }}_{1}$ was 0.04 and 0.03 respectively. For both estimators, doubling the sample size approximately halved the bias.

## Discussion

This paper has introduced a simple test of equal variance ratios (EVR), whose purpose is to determine when moderation of correlations and slopes are not equivalent. The test can be inverted to produce an approximate confidence interval for the ratio comparison of these two kinds of moderator effects. This test also may be extended easily to assessing whether the moderation of standardized and unstandardized regression coefficients are unequal.

Simulation results indicated that when EVR holds, Type I error rates are reasonably accurate but slightly high. Skew inflates Type I error rates somewhat, but not dramatically. When EVR does not hold, moderately large samples and effect-sizes are needed for high power, but HeV, HeEV, and unequal group sizes are not problematic for testing EVR or modeling the moderation of variance ratios. There is evidence that the EVR test has fairly high power, relative to the power to detect moderator effects.

Variance ratios for continuous moderators can be modeled via maximum likelihood methods, although no single model can deal with all forms of variance ratio moderation or HeV. The model presented here uses the log link for the standard deviation submodel and the Fisher link for the correlation submodel, with possibly different predictors in each submodel and, potentially, polynomial terms for the predictors. Bayesian estimation methods also may be used, but that extension is beyond the scope of this paper. When EVR holds and correlations are unmoderated, Type I error rates are somewhat too high for δ*_r1_* and slightly too high for θ_1_ unless sample sizes are over 200 or so. Skew inflates Type I error rates for δ*_r1_* but only slightly for θ_1_. For moderated variance ratios and correlations, maximum likelihood estimates are only slightly upward-biased for both δ*_r1_* and θ_1_, and in the usual fashion this bias decreases with increasing sample size. Moderately large samples and effect-sizes are needed for high power, but apparently no more so than for categorical moderators.

Tests of EVR for categorical moderators can be entirely dealt with using multi-groups SEM, and Mplus and the lavaan package in R are able to incorporate these tests via appropriate model comparisons. It also is possible to fit such models via scripts in computing environments such as SAS and SPSS possessing appropriate inbuilt optimizers. The SEM approach makes it possible to test complex hypotheses regarding the (non)equivalence of moderation of slopes and correlations, and to obtain a clear picture of both kinds of moderator effects. The online supplementary material for this paper includes a four-category moderator example where EVR holds for two pairs of categories but not for all four. In fact, the SEM approach elaborates conventional moderated regression into a combination of models for moderated slopes and moderated correlations. In principle it may be extended to incorporate tests for equality of tolerance across groups, which would enable modeling the moderation of semi-partial correlations.

All told, for categorical moderators the EVR test comes reasonably close to fulfilling the criteria of simplicity, availability, robustness and power. Considerable work remains to be done before the same can be said for continuous moderators. Nevertheless, the EVR test proposed here is highly relevant for both experimental and non-experimental research in mainstream psychology, and would seem to be a worthy addition to the researcher’s toolkit.

## Conflict of Interest Statement

The author declares that the research was conducted in the absence of any commercial or financial relationships that could be construed as a potential conflict of interest.
